# Direct-Acting Antiviral Agents in Prevention of Maternal–Fetal Transmission of Hepatitis C Virus in Pregnancy

**DOI:** 10.3390/pathogens13060508

**Published:** 2024-06-16

**Authors:** Christopher Hartley, Trung Van, Wikrom Karnsakul

**Affiliations:** 1 The Department of Pharmacy, The Johns Hopkins Hospital, Baltimore, MD 21287, USA; 2 Department of Molecular Pharmacology and Physiology, Morsani College of Medicine, University of South Florida, Tampa, FL 33602, USA; 3 Division of Pediatric Gastroenterology, Hepatology and Nutrition, Department of Pediatrics, Johns Hopkins University School of Medicine, Baltimore, MD 21287, USA

**Keywords:** hepatitis C, pregnancy, direct-acting-antiviral agents

## Abstract

Prior to the Food and Drug Administration approval of ledipaspavir/sofosbuvir (Harvoni^®^) in 2014, the treatment of hepatitis C was interferon plus or minus ribavirin. This treatment had low cure rates for hepatitis C virus and was teratogenic and therefore avoided in pregnant patients. Vertical transmission is the most common transmission of hepatitis C in pediatric patients, whereas medical equipment that was not properly cleaned and sterilized, blood products which were not checked (historically), sharing and reusing syringes and needles, and dialysis are the most common forms of hepatitis C transmission in adults. The treatment of pregnant women with direct-acting antivirals is important because the treatment of pediatric patients cannot begin until three years of age and does not always occur prior to the symptom development of hepatitis C. This review article will include glecaprevir/pibrentasvir (Mayvret^®^), sofosbuvir/velpatasvir (Epclusa^®^), and sofosbuvir/velpatasvir plus voxilaprevir (Vosevi^®^). We aim to review the teratogenic risk of direct-acting antivirals as well as currently published clinical trials and ongoing research on direct-acting antiviral hepatitis C treatment in pregnancy in this publication.

## 1. Introduction

Hepatitis C virus (HCV) affects 58 million people worldwide [1]. The most common genotype globally is genotype 1, which affects 83.4 million people. In the United States, genotype 1 is the most common genotype, and the highest risk of adverse outcomes is from genotype 3 [2]. The transmission of HCV most commonly occurs due to reusing or sharing syringes and needles and has increased exponentially due to the number of persons who inject intravenous drugs secondary to the Opioid Epidemic [3]. The two peaks for new cases of chronic hepatitis C can be seen in patients who are 20–39 years of age and 55–70 years of age [3]. 

There are an estimated 3.2 million pediatric and adolescent patients who have hepatitis C globally [1]. Fetal adverse effects which have been linked to HCV include fetal growth restriction as well as low birthweight. These complications set a neonate up for intrauterine fetal growth restriction, preterm birth, congenital anomalies, and the requirement of ventilator assistance, as well as a longer-term risk of hepatocellular carcinoma and liver disease [4,5,6,7,8,9]. Additionally, in 2019, there was a financial burden of $30,414 in excess cost to families with infants who had hepatitis C during their initial hospitalization [10]. 

Acute hepatitis progresses to chronic hepatitis after around 6 months post-exposure in patients who do not spontaneously clear the virus. Chronic hepatitis presents in about 80% of those who acquire HCV through vertical transmission [11]. Eventually, these pediatric patients with chronic HCV infection can experience mild liver disease in infancy and more severe liver injury later in life [12,13,14]. One center reported five cases where the pediatric patients required liver transplantation due to accelerated progression to severe liver diseases and complications, and two subsequently died [15]. As a result, any delay in the treatment of HCV in the pediatric patient can progress to chronic hepatitis, severe morbidity, and potentially mortality, particularly if a child loses their linkage to medical care or faces a delayed confirmation of diagnosis at any point after birth.

The most common cause for pediatric patients to contract hepatitis C is due to vertical transmission from a mother to the fetus. Transmission risk is increased if the mother is coinfected with human immunodeficiency virus (HIV) or has a high viral load [1,5]. For instance, the vertical transmission rate of HCV increases by 3–4 times when the mother is coinfected with HIV-1 [16]. 

The higher rate of transmission of HCV when coinfected with HIV-1 suggests a mode of co-transmission; yet, the mechanism of this mode is unknown [17]. However, both HCV and HIV infect peripheral blood mononuclear cells (PBMCs), and an increase in the HCV replication rate is observed in the PBMCs of patients coinfected with HCV and HIV [18]. This suggests some mode of interaction between HIV and HCV, which serves as a risk factor for vertical transmission and HCV infection in the fetus. HCV is found in the liver as well as in the monocytes and macrophages of infected persons due to the virus requiring negative-sense RNA to replicate [1,5,19].

**Figure 1:**
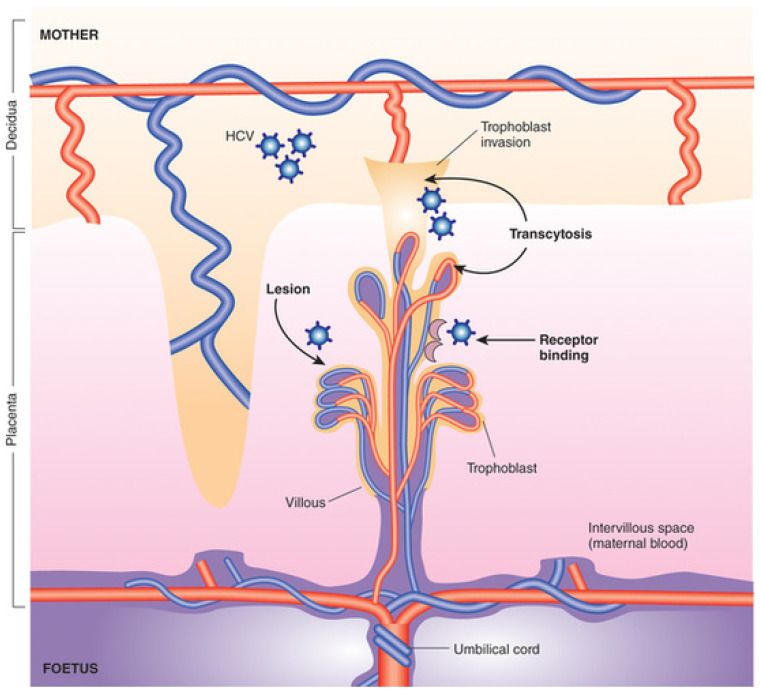
(Reprint of figure from Le Campion and colleagues [22]). Three potential modes of HCV fetal transmission: transcytosis, receptor binding, and lesion. The trophoblast, the epithelial layer of the placenta, consists of syncytiotrophoblast and cytotrophoblast. Cytotrophoblast serves as the inner layer of the trophoblast and eventually matures into the syncytiotrophoblast, which serves as the trophoblast’s outer layer. HCV from the mother can directly infect the fetus through cytotrophoblast transcytosis, binding to trophoblast’s HCV receptors, or injuries that affect the placental junction’s integrity [22].

Figure 1 demonstrates three potential modes of HCV fetal transmission, including transcytosis, receptor binding, and lesion. A healthy uterine decidua contains various immune cells including the macrophages, natural killer cells, and regulatory T cells [20]. The presence of these immune cells confers antiviral protection for the fetus, as they detect and eliminate microbial pathogens at an early phase. In addition, the activation and coordination of such immune cells differ depending on the infection. For example, TLR-3 stimulation followed by the trophoblast producing IFN-b and secretory leukocytes inhibits the vertical transmission of viral infection. Since HIV interferes with the functions of many of these immune cells [21], the maternofetal interface’s protection might be breached and allows the entry/co-transmission of HCV and eventually its replication and release. 

In addition to high maternal HCV viral load and having HIV, membrane rupture for more than 6 h before delivery and internal fetal monitoring (uterine or fetal scalp) have been reported to contribute to an increased risk of vertical HCV transmission [23]. The data suggest that HCV most likely infects the placenta directly, as the amniotic fluid of an infected mother rarely shows the presence of HCV. One study evaluating HCV RNA in amniotic fluid detected HCV RNA in 1/16 samples collected [24]. Syncytiotrophoblasts compose the placental barrier, where the exchange of substrates happens through transcytosis, and the permissiveness of these cells to various viral agents allows the infection of the placenta to take place (Figure 1). The essential components of HCV invasion require many HCV receptors and entry cofactors including tight junction proteins, such as occludin and claudin 6/9, LDLr, and SR-B1 [25,26]. The placenta expresses most of these entry factors for HCV, which explains how the HCV can directly infect the placenta, as demonstrated in a study that showed HCV infection in human cytotrophoblasts cultured in vitro [23]. Transmission may be most frequent during the peripartum period (estimated 40–50%) when there is blood–blood contact during delivery. In this case, the child will be born HCV-RNA negative, but detectable RNA levels are expected after the first 3 days of life. It is estimated that intrauterine transmission accounts for approximately 30% of cases, based on HCV-RNA positivity at or shortly after delivery [27]. The exact mechanisms by which intrauterine transmission occurs are not well understood but may include the ”trophoblast-mediated endocytosis of HCV and/or transcytosis of viral particles” [28]. Postpartum transmission via breastfeeding is rare, as the proportion of children acquiring HCV is similar among those who were breastfed compared to those who were not [29].

In an ideal setting, patients who wish to become pregnant should be tested for treatable viruses such as HCV and be appropriately treated for these viruses prior to becoming pregnant. However, this is not always the case. The Division of Viral Hepatitis Strategic Plan 2025 1.3.2. aims to increase coordination with centers to identify hepatitis B- and hepatitis C-infected mothers, increase testing, and promote linkage to care [30]. Hepatitis B reactivation is a risk for patients coinfected with hepatitis C. Patients need to be monitored for HBV prior to starting HCV treatment because HBV is not curable at this time and can lead to hepatic failure and death if not receiving therapy. 

Besides vertical transmission, another concern for HCV-infected mothers includes the risk of adverse pregnancy outcomes, which could lead to both short-term and long-term consequences for the child. Children born from HCV-infected mothers face a higher risk of preterm birth, lower birth weight, and smaller figures for gestational age [6,7,8,31]. Furthermore, studies also report the risk of developing gestational diabetes and intrahepatic cholestasis in HCV-infected pregnancy [32,33]. A proposal established in 2018 planned to perform a systemic review of pregnancy outcomes in pregnant mothers with HCV infection to characterize the short- and long-term risks associated with HCV-infected pregnancy; the results are to be published soon [34]. The treatment of HCV in children is not the aim of this study and is reviewed elsewhere [35].

Medications pharmacodynamically affect the patient not only in a positive way but also potentially in a neutral or even a negative way. Prior to the advent of direct-acting antivirals (DAAs), patients were treated with interferon (pegylated or not) plus or minus ribavirin therapy. Ribavirin is contraindicated in pregnancy, and if either partner is receiving ribavirin, then the prospective parents should wait at least six months prior to conception [36,37]. This is due to ribavirin’s long half-life as well as erythrocyte distribution, which can last up to 40 days after a single dose [38,39]. If patients or their partners are on ribavirin and get pregnant, then maternal and fetal outcomes should be reported to the ribavirin pregnancy registry. Ribavirin causes fetal death and fetal abnormalities in animal models [36].

The ribavirin registry was created by Merck, Genentedch, Aurobindo Pharma, Sandoz, Teva, Kadmon, and Zyydus and was utilized for 17 years. Data were submitted by the healthcare provider for the pregnant patient at enrollment, halfway through the pregnancy, and at delivery, and then for the infant at birth, 6 months, and 12 months. Birth defects were reviewed by the registry’s Birth Defect Evaluator and Scientific Advisory Board. Birth defects were defined as major structural or chromosomal abnormality or signs and symptoms within 6 years of age; exclusions were any defects attributed to prematurity. There was a total of 280 live births evaluated; 14 patients had defects, which ranged from physical defects such as ventricular septal defects, hip dysplasia, polydactyly, and deafness, to enzymatic G6PD deficiency in one patient. Sinclair and colleagues do discuss in their discussion section a major limitation of this study being challenges with loss to follow up, as well as challenges with how intensely patients were screened for these birth defects compared to the overall birth rates throughout the U.S. [37].

The FDA has approved DAAs for children aged three and older, which protects most patients from developing complications of HCV. However, cirrhosis and advanced fibrosis can still occur during childhood for patients with chronic hepatitis C if untreated [40]. Other challenges that are faced with pediatric patients include the palatability of the granules of medication, adherence with the assistance of caregivers, and the frequency of the lab tests for these patients. In terms of taking these medications post-partum, there is also the question of the risk of the DAAs sequestering into the breast milk. Interestingly, small studies have shown that pregnancy can cause the spontaneous resolution of hepatitis C viremia, which is thought to be due to a release of tolerance in hepatitis C-specific lymphocyte responses that develop while the patient is pregnant [41]. T cell-mediated response, specifically via CD8+ cytotoxic T lymphocytes, antagonizes and plays a major role in the early clearance of HCV [42]. As a result, the pre-emptive elimination of HCV prevents vertical transmission in pregnancy. However, in chronic HCV infection, these T cells become exhausted, and the production of HCV quasispecies allows for the virus to escape from T cell recognition. In addition, hemochorial placentas have other defense mechanisms, including the constitutive production of interferons, to confer antiviral protection. Rodent studies show that the constitutive expression of type III interferons, activated by the Alu SINE RNA, acts as a part of fetal innate immunity without causing obstetrical complications [43].

To date, therapy with DAAs is not recommended by the AASLD in pregnant patients with the hope to prevent vertical transmission due to the lack of safety and efficacy data [36]. We aim to discuss teratogenic effects of the DAAs, published literature, and current ongoing trials of DAAs in pregnant patients. We aim to review the teratogenic risk of direct-acting antivirals as well as currently published clinical trials and ongoing research on direct-acting antiviral hepatitis C treatment in pregnancy in this publication.

## 2. Teratogenic Effects of DAAs 

### 2.1. Pregnancy 

The U.S. Food and Drug Administration (FDA) approved pan-genotypical direct-acting antivirals (DAAs), including glecaprevir/pibrentasvir (Mayvret^®^), sofosbuvir/velpatasvir (Epclusa^®^), and sofosbuvir/velpatasvir plus voxilaprevir (Vosevi^®^) for the treatment of patients with DAA failure. Ledipasvir/sofosbuvir (Harvoni^®^) is still commonly used and covers genotypes 1, 4, 5, and 6. Ledipasvir/sofosbuvir (Harvoni) was originally FDA approved in 2014, sofosbuvir/velpatasvir (Epclusa^®^) was approved in 2016, and glecaprevir/piebrentasvir (Mavyret^®^) was originally approved in 2017 [44,45,46,47]. 

DAAs are categorized as pregnancy class B (classes range from A-D, then X, with A indicating that well-controlled studies do not show risk and X indicating that there is positive evidence of risk, where risks likely outweigh benefits) using the old pregnancy and lactation category system, meaning that animal reproduction studies have failed to demonstrate a risk to the fetus; however, there are no adequate and well-controlled studies in pregnant women [48]. Other class B medications using the old category system included prenatal vitamins, acetaminophen, and many other prenatal medications. Since the testing of medications in pregnant and lactating patients is difficult due to varying levels of risk to both mother and infant, registries are generally utilized instead to keep track of data of outcomes based on different types of exposure [49]. As of October 2023, there are 171 different pregnancy registries through the FDA of different medications and vaccinations [50]. The society for maternal–fetal medicine consult series updates on hepatitis C in pregnancy at the current time only recommends DAAs if the patient is enrolled in a clinical trial [51]. Interestingly, they discussed no difference seen in a meta-analysis of spontaneous vaginal delivery versus cesarian section and therefore did not recommend a cesarian section strictly for HCV transmission risk [51,52]. The society also recommends that mothers do not avoid breastfeeding, unless the nipples are cracked or bleeding, as breastmilk alone is not a common cause of HCV transmission [51].

DAAs work in three distinctive ways and work as a combination of two drugs with different mechanisms of action. NS5A inhibitors (example: Ledipasvir, pibrentasvir, velpatasvir) block the replication complex formation of lipoprotein within the Golgi apparatus, which prevents the transport and release of HCV out of the hepatocyte, where it can affect other hepatocytes. NS5B polymerase inhibitors (example: sofosbuvir) incorporate themselves into the RNA-dependent RNA polymerase, preventing incoming nucleotides from being added to the RNA chain. NS3/4 protease inhibitors (example: glecaprevir) prevent positive single-stranded ribonucleic acid (RNA) from the HCV from replicating itself into the negative single-stranded RNA within the hepatocytes, which prevents the HCV from multiplying [44,45,46,47]. 

A review article by Freriksen and colleagues discussed DAAs in pregnancy with three abstracts with the data to date [53]. The first publication was related to a phase 1 study by Chapell and colleagues regarding the use of ledipasvir plus sofosbuvir in pregnant patients. The phase 4 clinical trial will be discussed more in the active/not recruiting section of this publication [54]. The second article reports on accidental exposure to sofosbuvir and daclatasvir with or without ribavirin in pregnancy, where 11 pregnancies were exposed to DAAs. Only one patient had delivery that was complicated by post-partum hemorrhage but was not linked to DAA exposure [55]. The third study was an abstract from India reporting 15 pregnant patients who were treated with ledipasvir and sofosbuvir for hepatitis C. All patients achieved sustained virologic response at week 12 (SVR12), and all baby examinations were normal, indicating that treatment appeared to be safe for the fetus and effective for the mother [56].

Pharmacokinetically, the absorption and the risk of toxic effects of medications, especially in relation to the fetus, is a safety parameter to be cognizant of. An article from Pfeifer and colleagues evaluated the difference in transporters (P-glycoprotein (P-gp), breast cancer resistance protein (BCRP), or equilibrative nucleoside transporters 1 and 2 (ENT1, ENT2)) of DAAs into the placenta. They found that in pregnant patients, P-gp and ATP-binding cassette (ABCB1) efflux transporter genes were increased 3-fold during pregnancy, likely to decrease the amount of toxins and drugs being transported into the placenta. P-gp and BRCP interact with sofosbuvir, a DAA medications. In Pfeifer and colleagues’ research, they found that despite inhibiting P-gp and BRCP, there were no differences in the effect of sofosbuvir uptake. They also found that sofosbuvir is not involved in ENT1 or ENT2 [57]. Table 1 summarizes the pharmacokinetic changes and drug–drug interactions that occur with the DAAs during pregnancy. 

### 2.2. Lactation 

A review article by Freriksen and colleagues reviewed DAAs in pregnancy and lactation. They discussed how breastfeeding in infants is generally considered to present a low risk of transmission, unless the nipples are cracked [29,51,65]. Freriksen and colleagues reviewed available animal study data on the lactation transfer of drugs into milk, which was studied in rats [53] Glecaprevir had low overall transfer into milk, whereas the other DAAs had high percentages of transfer into the milk [53]. Some adverse effects were seen with patients’ offspring (lower birth weight), but this was likely due to them receiving four times the concentration of normal human dosages. This led to ledipasvir without ribavirin becoming a pregnancy class B medication [66]. However, as more post-marketing data comes out, it will be important to return to this information because of differences between human and animal studies. Differences in mammary gland anatomy as well as physiology, protein binding, protein-to-fat ratio, and intake–efflux transporters make it difficult to determine exact effects on patients [53]. 

## 3. Ongoing Clinical Trials 

When using Clinicaltrials.gov, then filtering using Hepatitis C as the disease, pregnancy as the other term, and DAAs as the intervention, there were 10 results. From these, all but one of the studies were removed due to having exclusion criteria regarding pregnant or lactating women. 

### 3.1. Currently Recruiting: (1)

TiP-HEPC (treatment in pregnancy for Hepatitis C (NCT05368974)) by the Taskforce for Global Health in Atlanta, Georgia is currently recruiting, with an estimated enrollment of 100 patients. This study’s primary outcome is to assess the safety of DAA treatment in mother–infant pairs with exposure to DAA medications during pregnancy from the registry. Secondary outcomes include describing the frequency and distribution of exposure as well as the effectiveness of transmission and HCV remission in mothers and their infants [67]. 

### 3.2. Active/Not Recruiting: (1)

The Treatment of Chronic Hepatitis C During Pregnancy with Sofosbuvir/Velpatasvir (NCT04382404) by the University of Pittsburgh in collaboration with Gilead Sciences and the Eunice Kennedy Shriver National Institute of Child Health and Human Development (NICHD) is a phase 1 interventional trial with an estimated enrollment of 10 patients. Patients will be enrolled between 20–30 weeks gestation if they have chronic hepatitis C viremia and will be treated with a 12-week course of sofosbuvir/velpatasvir [68].

Outcomes which will be reviewed include pregnancy and delivery outcomes, as well as neonatal hepatitis C viral loads, information regarding their birth, and neurodevelopmental assessments. Pharmacologic sampling also will be reviewed, which includes systematic exposure to velpatasbuvir, sofosbuvir, and an inactive metabolite, as well as intracellular sofosbuvir every three weeks [68]. 

Results from the interim abstract of the phase four clinical trial were presented at the AASLD meeting in November. The primary outcome was SVR12 and preterm birth (defined as less than 37 weeks of age). The majority of the patients were white (81%), and 17 patients completed all testing with SVR12. Of these 17 patients, 100% had SVR12. There were 15 patients who had HCV RNA testing performed at delivery, and all 15 of these patients had undetectable HCV RNA at the time of delivery. The results from 16 infants who went through all testing (delivery, 8 weeks, 6 months, 12 months) showed that 100% of them had undetectable HCV RNA. Of the 24 infants delivered, 3 of them were preterm births (median 38 weeks (range 33 + 5, 41 + 1)). A total of 2 of them were due to the premature rupture of membranes, and one was spontaneous preterm labor. Of all the serious adverse effects which were reported (n = 13), none were deemed related to the sofosbuvir/velpatasbuvir medication. 

## 4. Discussion

Current gaps in what we know in regard to the treatment of hepatitis C in pregnancy include treating prior to the 20-week gestational period that was performed in the current Gilead study; how to treat HIV+ coinfected patients, as those patients were removed from the STORC study; and whether these medications are safe in neonates, as this is an area that has not been evaluated, and current HCV dosing in pediatrics is only FDA approved in ages three and up [69]. 

More studies will need to be conducted to characterize the safety and efficacy profile of DAAs, specifically in pregnancy, because of the complex pregnancy-induced changes in drug absorption and metabolism. To date, only three abstracts in the world mention exposure to DAAs in a small sample size of pregnant women, either intentional or accidental [54,55,56]. However, DAAs do present as a promising treatment, as various combinations of DAAs offer synergistical effect with minimal to no teratogenic consequences. Potentially, treatment with DAAs might be initiated between the end of the second trimester and early in the third trimester to avoid any interference with the organogenesis process. Other studies have indicated that a composite score combining risk factors for HCV vertical transmission can potentially identify pregnant mothers whose rate of vertical transmission is high, weighing the risk–benefit ratio for interventions during antepartum care [70].

Various factors and aspects need to be considered when prescribing DAAs as a treatment for HCV-infected pregnant women. These factors consist of the safety and efficacy of DAAs on both the mother and child during pregnancy, the placental absorption and metabolism of DAAs, and the safety of DAAs during the lactation period. In animal studies, most DAAs have been observed to cross the placenta and become present in breast milk. However, various DAA combinations show high efficacy and safety profiles. 

## 5. Conclusions

Due to the relatively new options developed over nearly the past decade for the treatment of HCV, patients are now more likely to experience a cure. Pregnancy in HCV is overall still a large risk internationally, and treatment options for HCV in this patient population are still limited. In the new literature being published in regard to pan-genotypic DAAs (glecaprevir/pibrentasvir, sofosbuvir/velpatasvir, and sofosbuvir/velpatasvir plus voxilaprevir), they seem to be relatively safe options during pregnancy and did not result in vertical transmission. 

## Figures and Tables

**Table 1:** Pharmacokinetic changes in pregnancy and drug–drug interactions with DAAs.

	Changes in Pregnancy	DAA PK Information	Possible Drug–Drug Interactions with Pharmacokinetics and DAAs
**Absorption**	Delayed gastric emptying, prolonged transit time, reduced gastric activity [58,59]	Glecaprevir: Tmax 3–5 h, MSE increased 83–167% with meals [44] Ledipasvir: Tmax 4–4.5 h, no change in MSE with meals [47] Pibrentasvir: Tmax 5 h, MSE increased 40–114% with meals [44] Sofosbuvir: Tmax 0.5–1 h, MSE Increased 60% with meal [45,47] Velpatasvir: Tmax 3 h, MSE increased 34% with meal [45,46] Voxilaprevir: Tmax 4 h, MSE increases 100–400% with meal [46]	Proton pump inhibitors, antacids, H2 Receptor antagonists, GI motility agents, bile acid sequestrants [60]
**Distribution**	Apparent volume of distribution increases with highly lipophilic medications [58,61]	Glecaprevir: 97.5% protein bound [44] Ledipasvir: 99.8% protein bound [47] Pibrentasvir: >99.9% protein bound [44] Sofosbuvir: 61–65% protein bound [45,47] Velpatasvir: >90% protein bound [45,46] Voxilaprevir: 99% protein bound [46]	N/A
**Metabolism**	CYP3A4 activity increases (35–38% [62]), PGP, ENT1 and 2, and ABCB1 efflux transporters increase [57]	Glecaprevir: Secondary, CYP3A [44] Ledipasvir: Not CYP [47] Pibrentasvir: None [44] Sofosbuvir: Not CYP [45,47] Velpatasvir: CYP3A4 [45,46] Voxilaprevir: CYP3A4 [46]	CYP3A4 inhibitors (-azole antifungals, macrolide antibiotics, non-DPH calcium channel blockers, ritonavir, grapefruit juice) and CYP3A4 inducers (Rifampicin, phenytoin, barbituats, carbamazepine, corticosteroids, St. John’s wort) [63]
**Excretion**	Little information regarding changes in biliary excretion. Renal blood flow and glomerular filtration rate (GFR) increase [58].	Glecaprevir: Biliary [44] Ledipasvir: Majority biliary, minor renal (1%) [47] Pibrentasvir: Biliary [44] Sofosbuvir: Glomerular secretion, tubular secretion [45,47] Velpatasvir: Biliary [45,46] Voxilaprevir: Biliary [46]	Nephrotoxic medications (NSAIDs, aminoglycosides, vancomycin, ACE inhibitors, ionized contrast agents, diuretics) [64]

Legend: MSE, mean systemic exposure; H2 receptor antagonist, histamine-2 receptor antagonist; GI, gastrointestinal; CYP, cytochrome P; PGP, p-glycoprotein; ENT1/ENT2, equilibrative nucleoside transporters 1 and 2; ABCB1, ATP-binding cassette B1; -azole antifungal, fluconazole, itraconazole, voriconazole, Posaconazole; NSAID, non-steroidal anti-inflammatory; ACE inhibitor, angiotension-converting enzyme; N/A, Not-applicable.
